# Histopathologic analysis of stage pT1b kidney neoplasms for optimal surgical margins of nephron-sparing surgery

**DOI:** 10.1007/s12094-018-1845-0

**Published:** 2018-03-21

**Authors:** G. Li, Q. Luo, Z. Lang, Y. Li, A. Wang, K. Wang, Y. Niu

**Affiliations:** 1 Department of Urology, Tianjin Institute of Urology, The Second Hospital of Tianjin Medical University, No. 23, Pingjiang Rd, Tianjin, 300211 China; 2 Department of Pathology, Yuhuangding Hospital of Qingdao University, Yantai, Shandong China; 3 Department of Pathology, The People’s Hospital of Liaocheng, Liaocheng, Shandong China; 4 Department of Uropathology, Tianjin Institute of Urology, The Second Hospital of Tianjin Medical University, Tianjin, China; 5 Department of Urology, Yuhuangding Hospital of Qingdao University, Yantai, Shandong China

**Keywords:** Kidney neoplasms, Nephron-sparing surgery, Positive surgical margins, Peritumoral pseudocapsule, Multifocality

## Abstract

**Objective:**

To evaluate the pathological features and define the optimal surgical margins (SM) of nephron-sparing surgery (NSS) for kidney neoplasms 4–7 cm (stage pT1b) on preoperative imaging.

**Materials and methods:**

The retrospective study included 748 patients who were diagnosed stage pT1b renal tumors and underwent either radical nephrectomy (RN, *n* = 475) or NSS (*n* = 273) from January 2004 to March 2017. The tumor size, pathological subtype, Fuhrman grade, status of peritumoral pseudocapsule (PC) and tumor multifocality were recorded. The relationship between peritumoral PC and positive SM was calculated statistically by Fisher’s exact probability test.

**Results:**

The mean tumor diameter was 5.4 cm (range: 4.1–7.0 cm), 65 (8.7%) cases were discovered with multifocal lesions and 686 (91.7%) were surrounded with peritumoral PC in all 748 specimens. 57 (8.3%) of 686 cases were proved with tumor infiltrated beyond PC [infiltration (+)], and the presence of PC infiltration (+) was significantly correlated with positive SM (*p* = 0.016). The infiltrative depth of tumor cells into renal parenchyma beyond PC was all limited in 3 mm and the proportion of ≤ 1, 1–2 and 2–3 mm was 21.1% (12/57), 59.6% (34/57) and 19.3% (11/57), respectively.

**Conclusions:**

Our report indicates a 3 mm excisional margin is acceptable to ensure negative SM when operating NSS on stage pT1b kidney neoplasms.

## Introduction

Kidney neoplasms accounts for almost 3% of all malignant tumors [1], which includes variable kinds of pathological subtypes and different histopathological features. Nephron-sparing surgery (NSS) has been an optional manner for stage pT1a–1b (tumor diameter ≤ 7 cm) kidney tumors. Nevertheless, it is still controversial to confirm the optimal surgical margins (SM) for pT1b tumors [2, 3]. In the present study, we collected data from 748 cases of stage pT1b renal tumors and analyzed their pathological features retrospectively, especially the relationship between positive SM after NSS with the infiltrative situation of peritumoral pseudocapsule (PC). The main purpose of this study is to provide our experience and define the optimal SM when operating NSS for pT1b stage renal carcinomas.

## Materials and methods

### Patients

This retrospective study was approved by the ethical committee of the Second Hospital of Tianjin Medical University. 748 patients who were diagnosed stage pT1b renal carcinoma and underwent either RN (*n* = 475) or NSS (*n* = 273) from January 2004 to March 2017 in three institutional centers were enrolled in this study. All lesions in this research were unilateral, pathologically proved and without local or distant metastasis, hereditary tumors were excluded as well. Incorporated patients accepted preoperative ultrasonography of urinary system, abdominal computed tomography (CT) or magnetic resonance imaging (MRI) and chest X-ray to clarify tumor location, tumor size, TNM classification and the presence of metastatic lesions. A total of 527 (70.5%) males and 221 (29.5%) females were enrolled, with a male/female ratio of 2.38:1.00. The average age of patients was 60.2 (range: 26–88) years. 369 (49.3%) tumors were located in left kidneys and other 379 (50.7%) were in right (Table 1). Major clinical symptoms of 124 (16.6%) patients were gross hematuria, 85 (11.4%) were renal area pain, 49 (6.5%) were both gross hematuria and pain, other 490 (65.5%) cases had no obvious symptoms.

**Table 1:** Clinical and pathological characteristics of all patients

Characteristics	Value
No. of patients	748
Age (years)	
Range	26–88
Average	60.2
Sex	
Male	527 (70.5%)
Female	221 (29.5%)
Tumor location	
Left	369 (49.3%)
Right	379 (50.7%)
Tumor diameter (cm)	
Range	4.0–7.0
Average	5.4
Pathological subtype	
Clear cell	563 (75.3%)
Chromofobe	48 (6.4%)
Papillary	43 (5.7%)
Others	94 (12.6%)
Fuhrman grade	
1	152 (20.3%)
2	528 (70.6%)
3	47 (6.3%)
4	21 (2.8%)
Peritumoral pseudocapsule	
Absent	62 (8.3%)
Present	686 (91.7%)
Tumor multifocality	
Absent	683 (91.3%)
Present	65 (8.7%)

### Clinicopathological assessment

**Fig. 1:**
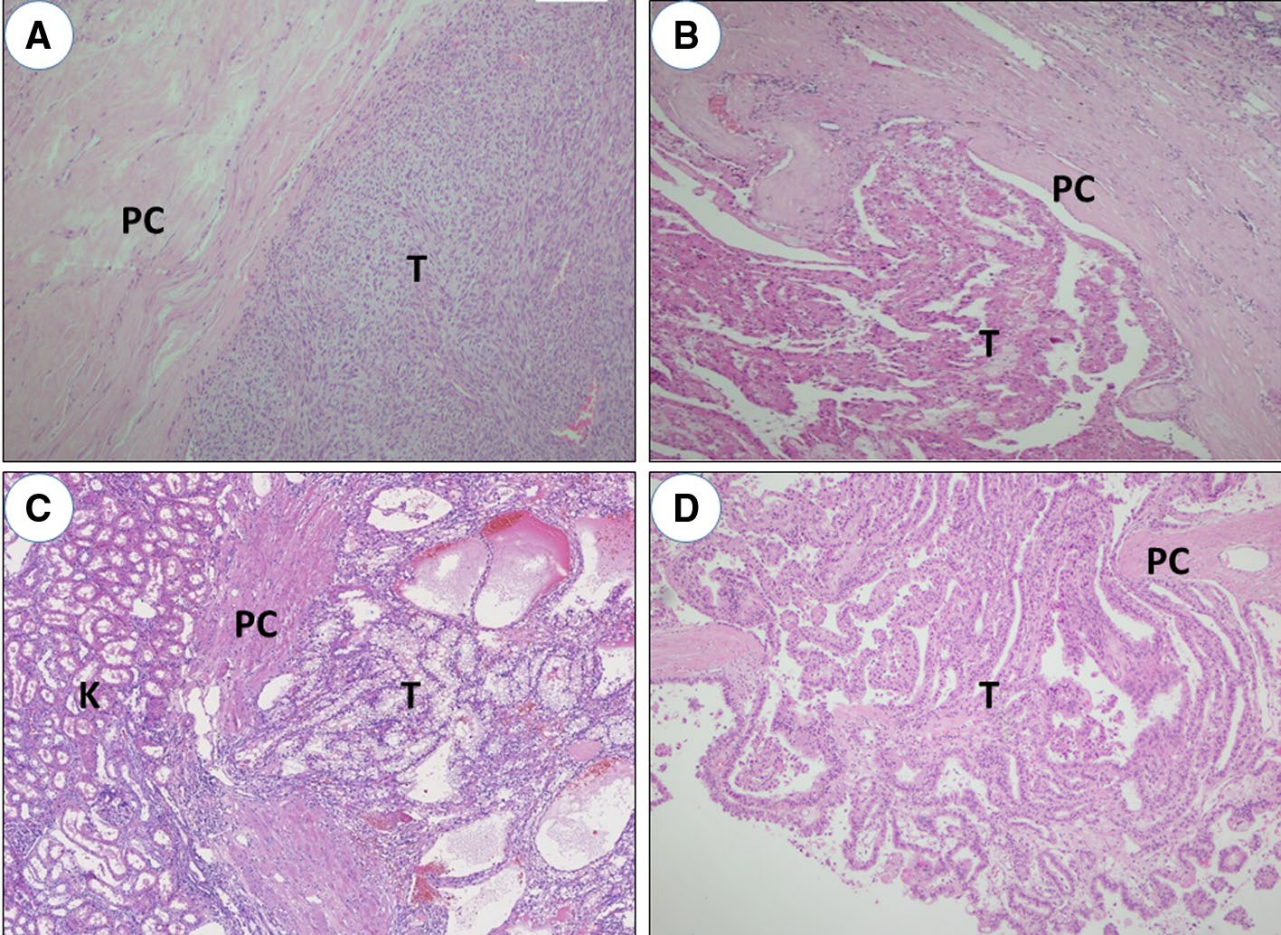
Representative figures of peritumoral PC (H&E × 100). **a**, **b** Peritumoral PC was intact and no tumor cells infiltrated beyond it;** c**,** d** tumor cells infiltrated beyond peritumoral PC. *PC* pseudocapsule, *T* tumor, *K* kidney

**Fig. 2:**
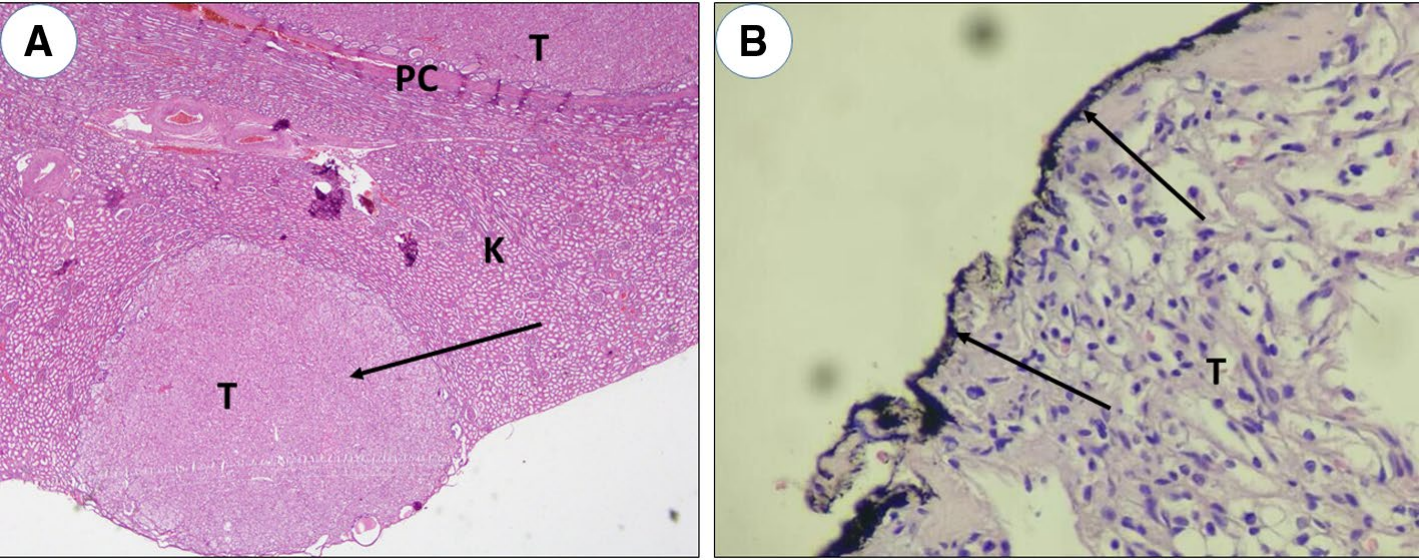
Representative figures of multifocal tumors and positive SM. **a** multifocal tumors by microscopic examination (H&E; × 100); **b** positive SM by pathological examination (ink dyed and H&E × 400). *SiM* surgical margins, *PC* pseudocapsule, *T* tumor, *K* kidney

The excisional margin of NSS was along the visible renal parenchyma, about 5–10 mm from tumor surface. For specimens of RN, we resected a 5–10 mm thickness of renal parenchyma around cancerous lesions in vitro and then performed pathological examination. The tumor size, pathological subtype, Fuhrman grade, peritumoral PC status and tumor multifocality were recorded and assessed. The highest representation of tumor–kidney interface of specimens was selected to standardize the material. Pathological data were done according to the 2004 WHO histopathological classification and 2010 AJCC TNM staging system. Peritumoral PC was defined as the presence of continuous fibrous tissue at the interface of tumor and adjacent renal parenchyma. PC status could be divided into 2 groups: Infiltration (−) means peritumoral PC was complete and no tumor cells infiltrated beyond it (Fig. 1a, b); Infiltration (+) means neoplastic cells infiltrated beyond peritumoral PC, even reached peritumoral normal renal parenchyma (Fig. 1c, d). The infiltrative depth of cancer cells in normal renal parenchyma beyond PC was also measured microscopically for each specimen. Tumor multifocality was defined as two or more lesions occurred in homolateral kidney with intervening normal renal parenchyma (Fig. 2a). Positive SM was certified by postoperative pathological examination (Fig. 2b).

### Statistical analysis

The Fisher’s exact probability test was used to compare the relationship between peritumoral PC and positive SM. A *p* < 0.05 was considered as statistical difference. Statistical analyses were performed using the Statistical Package for Social Sciences software version 20.0 (SPSS, Chicago, IL).

## Results

The clinical and pathological characteristics of patients are shown in Table 1. The mean tumor diameter was 5.4 (range: 4.1–7.0) cm. Based on the 2004 WHO classification, 563 (75.3%) cases were clear cell renal cell carcinoma (RCC), 48 (6.4%) were chromofobe RCC, 43 (5.7%) were papillary RCC and the remainders (12.6%) were other subtypes. Fuhrman grade I–IV was found in 152 (20.3%), 528 (70.6%), 47 (6.3%) and 21 (2.8%) patients, respectively. Among 748 specimens, 686 (91.7%) cases were surrounded with peritumoral PC and 65 (8.7%) cases were discovered with multifocal masses. 20 (30.8%) multifocal lesions were discovered via preoperative imaging and other 45 (69.2%) through postoperative pathological test.

57 (8.3%) of 686 specimens were proved with PC infiltration (+), and the infiltrative depth of tumor cells into peritumoral renal parenchyma beyond PC was all limited in 3 mm from primary tumor surface. The infiltrative distance in renal parenchyma of ≤ 1, 1–2 and 2–3 mm was 12 (21.1%), 34 (59.6%) and 11 (19.3%) cases, respectively (Table 2).

**Table 2:** The infiltrative depth of tumor cells in peritumoral normal renal parenchyma beyond PC

Infiltrative depth	Value
No. of patients	57
≤ 1 mm	12 (21.1%)
1–2 mm	34 (59.6%)
2–3 mm	11 (19.3%)
> 3 mm	0 (0)

*PC* pseudocapsule

Peritumoral PC was detected in 254 (93.0%) of 273 patients who underwent NSS. In these 254 specimens, the situation of PC infiltration (+) and (−) were found in 19 (7.5%) and 235 (92.5%) cases separately. Positive SM was discovered in 8 (2.9%) of 273 patients pathologically and all of the 8 kidney neoplasms were surrounded with PC. Statistical analysis shows the presence of PC infiltration (+) was significantly correlated with positive SM (*p* = 0.016), as shown in Table 3.

**Table 3:** The relationship of PC infiltration and positive SM

Infiltration	SM		*p* value
	(+)	(−)	
(+)	3	16	
(−)	5	230	0.016

*PC* pseudocapsule, *SM* surgical margins

## Discussion

Kidney neoplasms occupy approximately 3% of reported human tumors worldwide, and the morbidity in developed countries is higher than developing countries [1, 4]. Surgical resection is the dominant treatment for localized renal neoplasms. With the improvement of surgical techniques and an increasing awareness of the long-term postoperative renal function, NSS has been widely accepted by urologists and applied in renal carcinomas 4 cm or less in recent years. Compared with RN, NSS is preferable to preserve more renal parenchyma and obtain better oncologic outcomes through long-term follow-up [5, 6]. Leibovich et al. [7] retrospectively compared outcomes of 91 stage pT1b patients treated with NSS and 841 pT1b patients with RN, and concluded that no significant differences of cancer-specific survival or distant metastases-free survival between two groups. Such similar conclusions were proved in other literatures as well [8–10].

Compared with RN, NSS takes advantage of renal functional preservation, oncological control and maximizing prevention of tumor recurrence. Whether positive SM is significantly correlated with long-term risk of local recurrence and distant organic metastasis still remains controversial [11–14]. A number of researches have been conducted to define an optimal excisional margin. Zucchi et al. [15] proposed a 10 mm margin of normal-appearing parenchyma in operating NSS was enough for pT1b kidney cancer to ensure negative SM and decrease the risk of tumor recurrence. While, some authors considered such resection distance might lead to an unnecessary overexcision of normal parenchyma and increase the incidence of surgical complications, such as postoperative bleeding, damage of urinary collecting system and hilar vessels. Sutherland [16] stated a margin width of normal renal parenchyma less than 5 mm during PN for stages T1-2N0M0 RCC was suitable and safe to ensure a negative SM. Nevertheless, Akcetin et al. [17] suggested a 2 mm surgical distance for tumors < 5 cm was safe enough on survival after NSS, and an additional resection was unnecessary and irrelevant with postoperative progression. Berdjis et al. [18] explored 121 patients with NSS and concluded that the width of resection margin did not have influence on the risk of tumor recurrence. Chen et al. [19] retrospectively analyzed 87 specimens of T1b RCC and found 34 (39%) cases had extra-PC cancerous lesions. The distance of such lesions distributed in 1, 2, 3 mm was 11, 21 and 7%, respectively, and they recommended a 4 mm optimal SM. In our report, we found the presence of tumor cells infiltrated beyond peritumoral PC was significantly correlated with positive SM (*p* = 0.016) and all extra-PC lesions were within the width of 3 mm from primary tumor surface as well. Accordingly, a resection margin of 2 mm or less is not appropriate, especially for tumors which infiltrate into renal parenchyma beyond PC. To ensure a negative SM, we recommend an excisional distance of 3 mm at least is reliable.

The dominant concerns against NSS derive from the presence of multifocal neoplasms, which was recognized by either preoperative imaging or postoperative pathological examination. In a review paper published earlier this year, the authors indicated that removing all discernible tumors was likely more important than excess SM width [20]. Lee et al. [21] found that tumor multifocality existed in 5.3% (57/1071) RN specimens and only 33.3% (19/57) could be discovered on preoperative imaging, undetected occult multifocality was present in 3.5% (38/1071) RCC patients. Nevertheless, Whang et al. [22] reported an obvious higher proportion, of which 25% (11/44) of RCC demonstrated pathological multifocality and multifocal rate was independent of the size of primary tumors. In a meta-analysis of 1180 patients who underwent NSS, authors discovered the incidence of multifocal renal lesions was approximately 15% and it depended on tumor size, histology and stage [23]. The discrepancy of reported multifocal incidences was potentially caused by the difference of pathological methodology. In our study, the frequency of tumor multifocality was 8.7% (65/748), which was in accordance with reported ratio. Small multifocal lesions were easily visualized through preoperative imaging examination (only 13.1–44% cases were recognized [24–26]), including abdominal ultrasound, CT or MRI. In the present study, preoperative imaging detected 20 (30.8%) multifocal lesions only, other 45 (69.2%) were discovered via pathological test. The missing satellites might lead to tumor recurrence or positive SM [27], while it is still unclear whether multifocal foci is capable of progressing to local or distant metastatic tumors. A retrospective research revealed that compared with unifocal tumor, no statistic difference of overall survival, disease-free survival and disease-free rate was found in multifocal group after a 3 and 5 years’ follow-up [21].

Here, we provided our experience and advice to avoid postoperative positive SM and tumor recurrence for NSS on stage pT1b kidney tumors. First, the safe distance of SM away from tumor which is infiltrated into renal parenchyma should better be more than 3 mm. For tumors surrounded with intact peritumoral PC, an intraoperative frozen-section examination is not necessary and propositional (EAU guidelines). Moreover, it is important to inspect carefully whether multifocal neoplasms exist around the primary tumor according to preoperative imaging examination and intraoperative visual field. If so, all multifocal lesions should be excised entirely during surgery to prevent residual cancer cells.

There were also some limitations, including that our research was a retrospective analysis and non-randomized design which could potentially decrease the level of evidence. Besides, the pathological data were collected from different medical institutions over a long period and the procedures of handling specimens were not uniform. Despite these limitations, we had faith in the usefulness of our results for surgeons to perform optimal SM during operation. These findings need to be validated in future prospective studies.

## Conclusion

Our analysis about stage pT1b kidney neoplasms showed that positive SM was statistically related to the status of peritumoral PC and the infiltrative depth of tumor cells in normal renal parenchyma beyond PC was all limited in 3 mm. Thus, a 3 mm resection margin of renal parenchyma is appropriate to ensure negative SM. When operating NSS on stage pT1b patients, we recommend careful pre- and intra-operative inspection for multifocal tumors to prevent residual cancer cells and tumor recurrence.

## Abbreviations

NSS: Nephron-sparing surgery
RN: Radical nephrectomy
PC: Pseudocapsule
SM: Surgical margins
CT: Computed tomography
MRI: Magnetic resonance imaging
